# Supplementary material for: Spatially distinct epithelial and mesenchymal cell subsets along progressive lineage restriction in the branching embryonic mammary gland
Source: EMBO J. 2024 May 17;43(12):3. doi: 10.1038/s44318-024-00115-3 (PMC11183262; doi:10.1038/s44318-024-00115-3)
Supplement: Supplementary file 4 — Expanded View Figures [file 44318_2024_115_MOESM4_ESM.pdf]

Expanded View Figures

Figure EV1. Related to Fig. 1. Lineage-committed cells exist in early MG development.

(**A**) Representative FACS dot plots of the gating strategy used to sort E15.5 and P0 epithelial and mesenchymal cells. (**B**) UMAP plots of embryonic MECs and surrounding mesenchymal cells isolated by scRNA-seq at E13.5, E14.5, E15.5 and P0. Cells are color-coded by cluster. (**C**) UMAP plot of embryonic MECs isolated at E15.5 after subset analysis of all MECs (including proliferative cells shown in light blue). (**D**) Violin plot representation of the cell cycle score in each mammary epithelial cluster at E15.5 ($n = 430$ cells analyzed). (**E**) Heatmap showing the expression of genes specific for each cell cluster at E15.5. Each column is color-coded according to the cell cluster from (**B**). (**F**) UMAP plots from (**C**) showing the expression of specific luminal (*Krt8* and *Krt18*) and basal (*Krt5* and *Trp63*) genes commonly used to distinguish adult LCs and BCs but unable to discriminate distinct cell clusters at E15.5. (**G**) Box plots illustrating the log2 fold change of the luminal/basal score ratio in each cluster. $n = 22$ cells at E13.5; $n = 28$ cells at E14.5; $n = 98$ basal-like cells, $n = 199$ hybrid cells and $n = 86$ luminal-like cells at E15.5; $n = 19$ basal cells, $n = 140$ LP cells and $n = 39$ ML cells at P0. Statistical significance was assessed with Wilcoxon test. Lower and upper hinges correspond to the first and third quartiles. The upper whisker extends from the hinge to the largest value no further than 1.5 * IQR from the hinge (where IQR is the inter-quartile range, or distance between the first and third quartiles). The lower whisker extends from the hinge to the smallest value at most 1.5 * IQR of the hinge. Data beyond the end of the whiskers are called "outlying" points and are plotted individually.

© The Author(s)

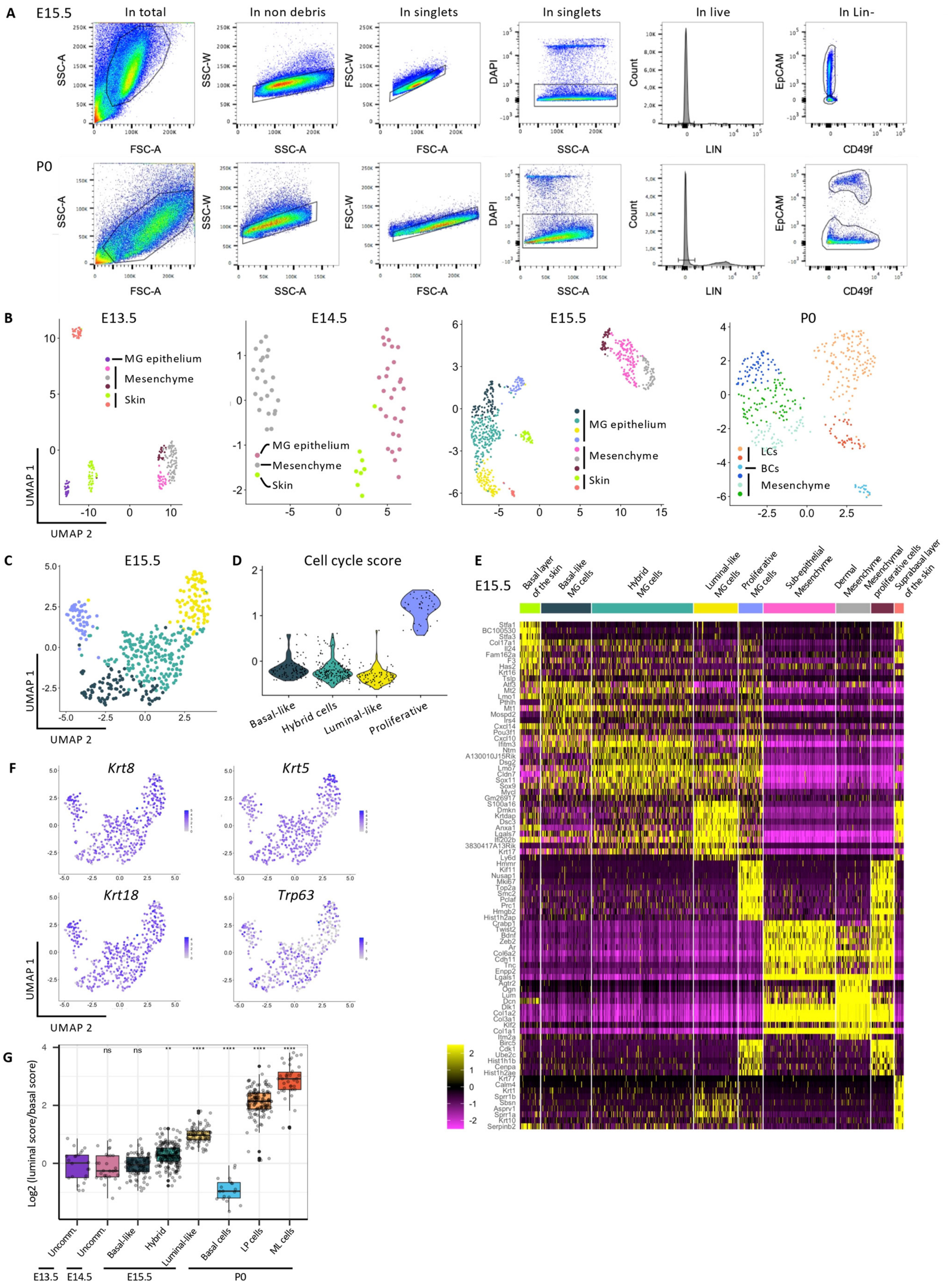

A
E15.5
In total
In non debris
In singlets
In singlets
In live
In Lin-
SSC-A
SSC-W
FSC-W
DAPI
Count
EpCAM
FSC-A
SSC-A
FSC-A
SSC-A
LIN
CD49f
P0
B
E13.5
E14.5
E15.5
P0
MG epithelium
Mesenchyme
Skin
LCs
BCs
UMAP 1
UMAP 2
C
E15.5
D
Cell cycle score
Basal-like
Hybrid cells
Luminal-like
Proliferative
E
E15.5
Basal layer of the skin
Basal-like MG cells
Hybrid MG cells
Luminal-like MG cells
Proliferative MG cells
Sub-epithelial Mesenchyme
Dermal Mesenchyme
Mesenchymal proliferative cells
Suprabasal layer of the skin
F
Krt8
Krt5
Krt18
Trp63
G
Log2 (luminal score/basal score)
ns
ns
**
****
****
****
****
Uncomm.
Uncomm.
Basal-like
Hybrid
Luminal-like
Basal cells
LP cells
ML cells
E13.5
E14.5
E15.5
P0

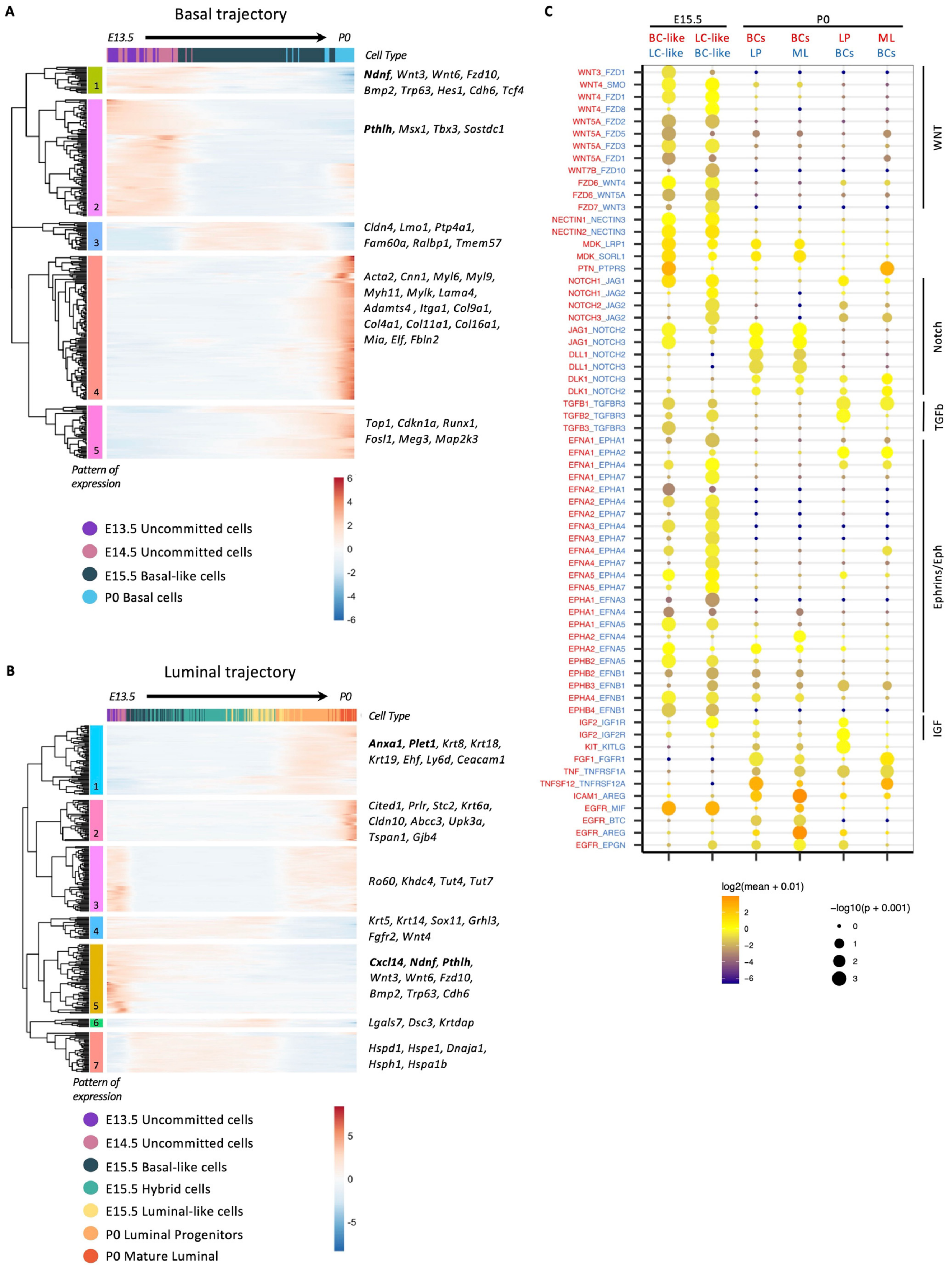
A
Basal trajectory
E13.5
P0
Cell Type
Ndnf, Wnt3, Wnt6, Fzd10, Bmp2, Trp63, Hes1, Cdh6, Tcf4
Pthlh, Msx1, Tbx3, Sostdc1
Cldn4, Lmo1, Ptp4a1, Fam60a, Ralbp1, Tmem57
Acta2, Cnn1, Myl6, Myl9, Myh11, Mylk, Lama4, Adamts4 , Itga1, Col9a1, Col4a1, Col11a1, Col16a1, Mia, Elf, Fbln2
Top1, Cdkn1a, Runx1, Fosl1, Meg3, Map2k3
Pattern of expression
E13.5 Uncommitted cells
E14.5 Uncommitted cells
E15.5 Basal-like cells
P0 Basal cells
B
Luminal trajectory
E13.5
P0
Cell Type
Anxa1, Plet1, Krt8, Krt18, Krt19, Ehf, Ly6d, Ceacam1
Cited1, Prlr, Stc2, Krt6a, Cldn10, Abcc3, Upk3a, Tspan1, Gjb4
Ro60, Khdc4, Tut4, Tut7
Krt5, Krt14, Sox11, Grhl3, Fgfr2, Wnt4
Cxcl14, Ndnf, Pthlh, Wnt3, Wnt6, Fzd10, Bmp2, Trp63, Cdh6
Lgals7, Dsc3, Krtdap
Hspd1, Hspe1, Dnaja1, Hsph1, Hspa1b
Pattern of expression
E13.5 Uncommitted cells
E14.5 Uncommitted cells
E15.5 Basal-like cells
E15.5 Hybrid cells
E15.5 Luminal-like cells
P0 Luminal Progenitors
P0 Mature Luminal
C
E15.5
P0
BC-like LC-like
LC-like BC-like
BCs LP
BCs ML
LP BCs
ML BCs
WNT3_FZD1
WNT4_SMO
WNT4_FZD1
WNT4_FZD8
WNT5A_FZD2
WNT5A_FZD5
WNT5A_FZD3
WNT5A_FZD1
WNT7B_FZD10
FZD6_WNT4
FZD6_WNT5A
FZD7_WNT3
NECTIN1_NECTIN3
NECTIN2_NECTIN3
MDK_LRP1
MDK_SORL1
PTN_PTPRS
NOTCH1_JAG1
NOTCH1_JAG2
NOTCH2_JAG2
NOTCH3_JAG2
JAG1_NOTCH2
JAG1_NOTCH3
DLL1_NOTCH2
DLL1_NOTCH3
DLK1_NOTCH3
DLK1_NOTCH2
TGFB1_TGFBR3
TGFB2_TGFBR3
TGFB3_TGFBR3
EFNA1_EPHA1
EFNA1_EPHA2
EFNA1_EPHA4
EFNA1_EPHA7
EFNA2_EPHA1
EFNA2_EPHA4
EFNA2_EPHA7
EFNA3_EPHA4
EFNA3_EPHA7
EFNA4_EPHA4
EFNA4_EPHA7
EFNA5_EPHA4
EFNA5_EPHA7
EPHA1_EFNA3
EPHA1_EFNA4
EPHA1_EFNA5
EPHA2_EFNA4
EPHA2_EFNA5
EPHB2_EFNA5
EPHB2_EFNB1
EPHB3_EFNB1
EPHA4_EFNB1
EPHB4_EFNB1
IGF2_IGF1R
IGF2_IGF2R
KIT_KITLG
FGF1_FGFR1
TNF_TNFRSF1A
TNFSF12_TNFRSF12A
ICAM1_AREG
EGFR_MIF
EGFR_BTC
EGFR_AREG
EGFR_EPGN
WNT
Notch
TGFb
Ephrins/Eph
IGF
log2(mean + 0.01)
−log10(p + 0.001)

© The Author(s)

◀ **Figure EV2. Related to Fig. 1. Pseudotime ordering identifies genes associated with early luminal and basal differentiation and changes in ligand-receptor interaction pairs between basal-like and luminal-like at early and late developmental timepoints.**

(**A**, **B**) Heatmaps illustrating genes exhibiting a differential pattern of expression along the pseudotime (from E13.5 to P0) towards the basal lineage (**A**) or the luminal lineage (**B**). Genes (rows) are clustered based on the dendrogram on the left and color-coded by their expression levels (from blue to red). The gene expression levels were smoothed using the generalized additive model (GAM) and scaled by row. Genes of interest are indicated on the right. Each set of genes with a specific pattern is color-coded on the left: 5 distinct patterns in the basal lineage (**A**) and 7 unique patterns in the luminal lineage (**B**). (**C**) CellPhoneDB analysis showing predicted ligand-receptor interactions between the two epithelial populations at E15.5, basal-like and luminal-like cells (left side of the dotplot), and between the three epithelial populations at P0; BCs, LP and ML cells (right side of the dotplot) (P value < 0.01). Permutation test was used for statistical analysis.

© The Author(s)

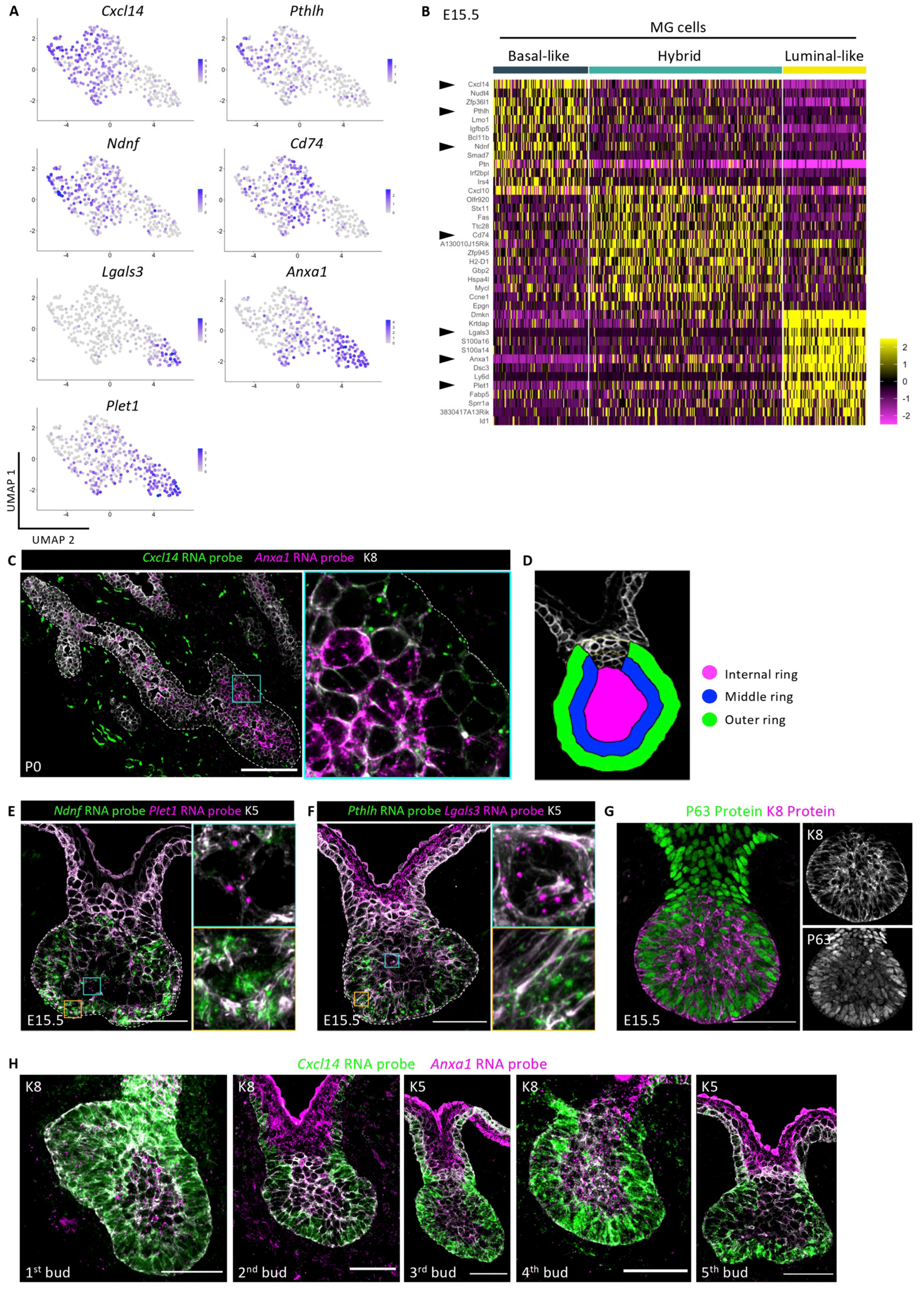
A
Cxcl14
Pthlh
Ndnf
Cd74
Lgals3
Anxa1
Plet1
UMAP 1
UMAP 2
B
E15.5
MG cells
Basal-like
Hybrid
Luminal-like
Cxcl14
Nudt4
Zfp36l1
Pthlh
Lmo1
Igfbp5
Bcl11b
Ndnf
Smad7
Ptn
Irf2bpl
Irs4
Cxcl10
Olfr920
Stx11
Fas
Ttc28
Cd74
A130010J15Rik
Zfp945
H2-D1
Gbp2
Hspa4l
Mycl
Ccne1
Epgn
Dmkn
Krtdap
Lgals3
S100a16
S100a14
Anxa1
Dsc3
Ly6d
Plet1
Fabp5
Sprr1a
3830417A13Rik
Id1
C
Cxcl14 RNA probe Anxa1 RNA probe K8
P0
D
Internal ring
Middle ring
Outer ring
E
Ndnf RNA probe Plet1 RNA probe K5
E15.5
F
Pthlh RNA probe Lgals3 RNA probe K5
E15.5
G
P63 Protein K8 Protein
K8
P63
E15.5
H
Cxcl14 RNA probe Anxa1 RNA probe
K8
K5
1st bud
2nd bud
3rd bud
4th bud
5th bud

 © The Author(s)

Figure EV3. Related to Fig. 2. Identification of novel genes that distinguish lineage-biased embryonic mammary cells.

(**A**) UMAP plots showing the expression levels of selected basal (*Cxcl14*, *Pthlh* and *Ndnf*), hybrid (*Cd74*) and luminal (*Lgals3, Anxa1* and *Plet1*) genes at E15.5. (**B**) Heatmap illustrating the expression of genes specific for each MEC cluster at E15.5. Each column is color-coded according to the cell cluster from Fig. 1B. Black arrowheads indicate genes used in RNAscope experiments. (**C**) Representative section of a mammary duct at P0 showing the expression of *Cxcl14* (in green) and *Anxa1* (in magenta) detected by RNAscope and of K8 by IF (in white). The white dotted line delineates the BM ($n = 3$). (**D**) Optical section of a mammary bud at E15.5 illustrating the ROIs: outer ring (in green), middle ring (in blue) and internal ring (in magenta) used for the quantitative analysis. (**E**, **F**) Representative sections of embryonic mammary buds at E15.5 showing the expression of *Ndnf* (basal gene, in green) and *Plet1* (luminal gene, in magenta) (**E**) or *Pthlh* (basal gene, in green) and *Lgals3* (luminal gene, in magenta) (**F**), detected by RNAscope and immunostained with antibodies anti-K5 (in white) ($n = 2$). (**G**) Single optical section showing the expression of the luminal epithelial marker K8 (in magenta), and the basal epithelial marker P63 (in green) in an embryonic mammary bud at E15.5. K8 and P63 are co-expressed by all MECs at E15.5. (**H**) Representative sections of the 5 different embryonic mammary buds (#1 to #5) at E15.5, showing the conserved expression of *Anxa*1 (in magenta) and *Cxcl14* (in green) detected by RNAscope. Anti-K5 or anti-K8 immunostaining delineate the mammary bud epithelium (in white) ($n = 2$). Data information: scale bars: 100 μm in (**C**) and 50 μm in (**E**–**H**).

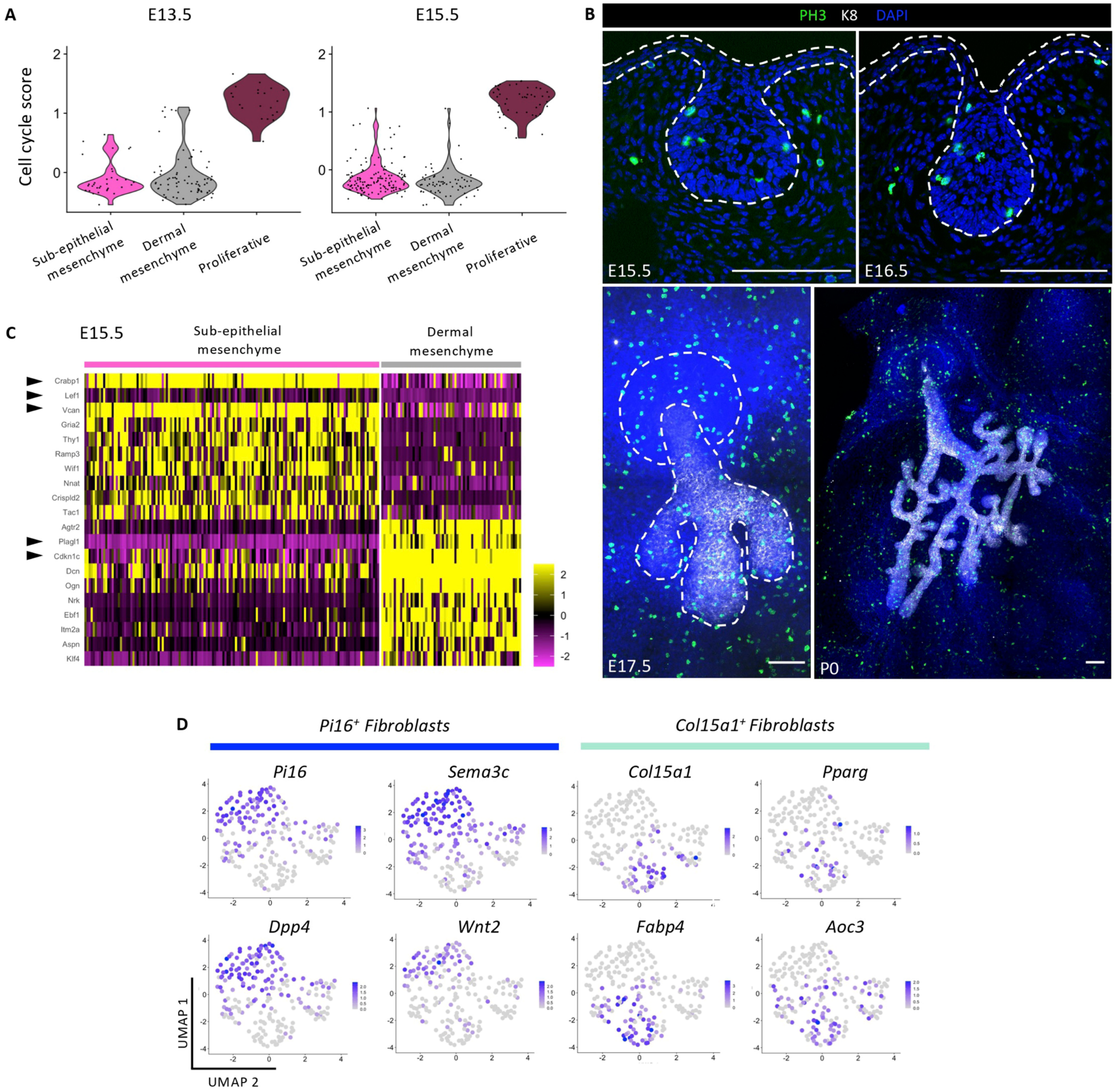

Figure EV4. Related to Fig. 3. The heterogeneity of mesenchymal cells increases at birth.

(**A**) Violin plots representing the cell cycle score in each mammary mesenchymal cluster at E13.5 and E15.5. $n = 129$ cells at E13.5; $n = 252$ cells at E15.5. (**B**) Representative sections of mammary bud at E15.5 and E16.5 and whole-mount staining at E17.5 and P0 showing PH3^{+} cells (in green), K8 (in white) and DAPI (in blue) ($n = 2$). Dotted lines delineate the BM (in white). (**C**) Heatmap illustrating the expression of genes specific for each mesenchymal cluster at E15.5. Each column is color-coded according to the cell cluster from Fig. 3A. The black arrowheads indicate the genes that were further investigated for their specific expression in sub-epithelial or dermal mesenchyme. (**D**) UMAP plots from Fig. 3A illustrating the expression of cluster-specific genes in mesenchymal cells at P0. Scale bars: 100 μm in (**B**).

 © The Author(s)

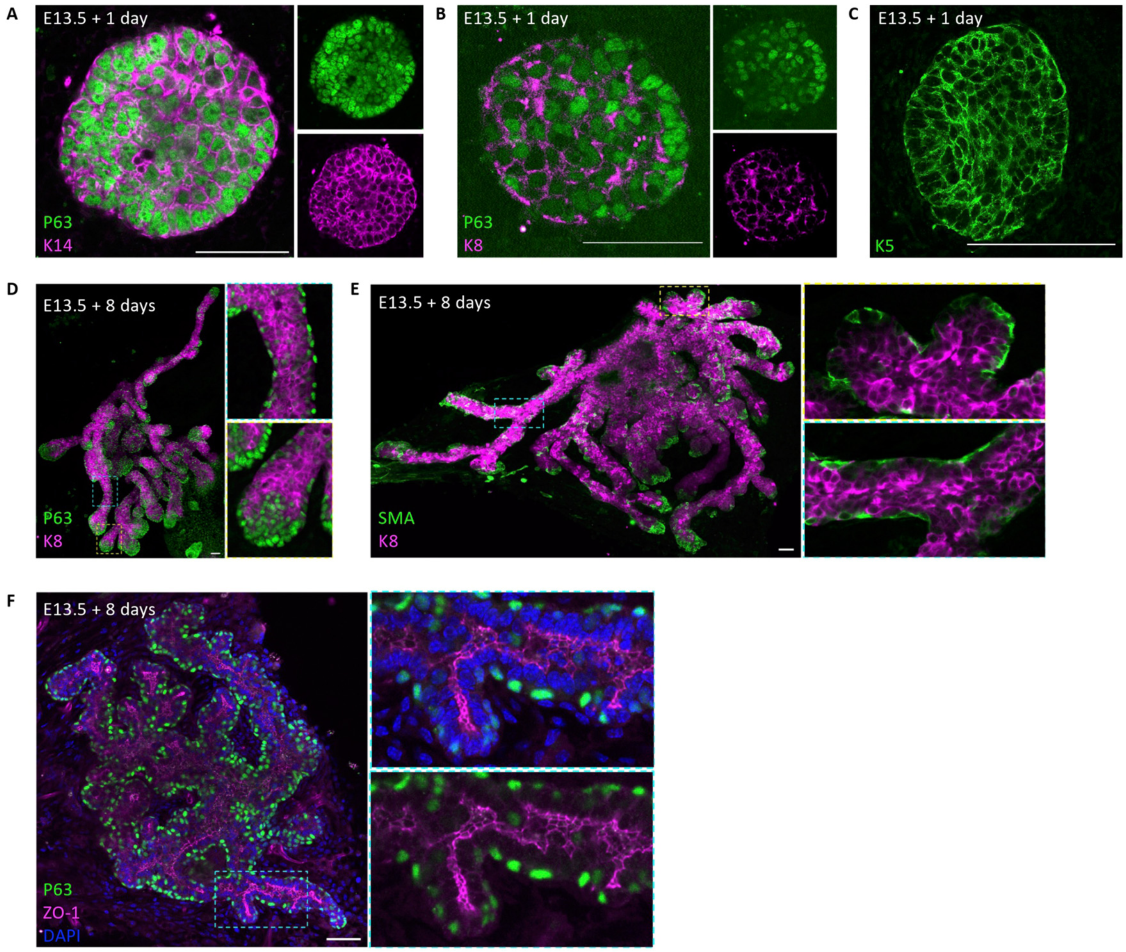

Figure EV5. Related to Fig. 5. Mammary bud *ex vivo* cultures recapitulate embryonic mammary morphogenesis and epithelial lineage segregation.

(**A–C**) Representative images of mammary embryonic buds dissected at day E13.5 and cultured ex vivo for 1 day, immunostained for the following lineage markers: P63 (in green) and K14 (in magenta) (**A**), P63 (in green) and K8 (in magenta) (**B**), and K5 (in green) (**C**). (**D–F**) Representative images of mammary embryonic buds dissected at day E13.5 and cultured ex vivo for 8 days, immunostained for the following lineage and polarity markers: P63 (in green) and K8 (in magenta) (**D**), α-SMA (in green) and K8 (in magenta) (**E**), and P63 (in green) and ZO-1 (in magenta) (**F**). Data information: scale bars: 50 µm (in **A–C**), 100 µm (in **D–F**) ($n = 3$).
